# m6A-related genes of peripheral white blood cell in spinal cord injury as potential targets for prognosis and treatment

**DOI:** 10.3389/fmed.2025.1544719

**Published:** 2025-04-09

**Authors:** Mingran Luo, Qian Wang, Jian Chen, Guoyong Yin

**Affiliations:** Department of Orthopedics, The First Affiliated Hospital of Nanjing Medical University, Nanjing, China

**Keywords:** spinal cord injury (SCI), N6-methyladenosine (m6A), white blood cells (WBC), peroxisome proliferator-activator receptor gamma (PPARG), adenylate kinase 5 (AK5), MRI

## Abstract

**Objective:**

Spinal cord injury (SCI) is a destructive neurological and pathological state that causes major motor, sensory, and autonomic dysfunction. N6-methyladenosine (m6A) is a reversible RNA modification implicated in various biological processes. However, few studies have examined m6A expression in patients with SCI. We explored the prognostic value of m6A-related genes as potential biomarkers in SCI to establish a set of accurate diagnostic and prognostic prediction models.

**Methods:**

Differentially expressed analysis and weighted gene co-expression network analysis (WGCNA) was used to explore m6a related modules and hub genes. KEGG and GO analyses was utilized to explore the potential role of these hub genes. Gene expression was verified in single-cell data. The correlation of m6A related gene with spinal cord injury severity was explored.

**Results:**

We found 289 SCI-related and five m6A-related candidate genes with high SCI correlation and high differential expression in the publicly available dataset, GSE151371. These genes are also involved in long-chain fatty acid binding. Early SCI was accompanied by significant immune cell infiltration. Simultaneously, infiltrating immune cells and the innate immune system have a strong cellular interaction, which gradually decreases over time. The number of PPARG-positive cells also increases after SCI. The comparatively higher expression of PPARG and lower expression of AK5 in white blood cells (WBCs) correlates with severity of SCI.

**Conclusion:**

Our integrated analysis illustrates the hub genes involved in SCI, which can be prognostic markers. Further understanding of the functions of the identified SCI hub genes may provide deeper insights into the molecular mechanisms of SCI.

## Introduction

Spinal cord injury (SCI) is a pathological and neurological condition associated with severe motor, sensory, and autonomic dysfunctions (1). A cascade of damaging processes, including ischaemia, oxidative stress, inflammatory events, apoptotic pathways, and locomotor dysfunction, are involved in its acute and chronic stages (2).

Depending on their phenotype, macrophages and microglia can have either positive or negative effects following spinal cord injury. In addition to playing a crucial role in inflammatory reactions, infections, apoptotic cells, and tissue debris are recognised, engulfed, and degraded by macrophages and microglia. They encourage remyelination and axonal regrowth by eliminating the inhibition of myelin components and cellular detritus (3). However, the lack of an effective method for cholesterol clearance in the central nervous system leads to the excessive accumulation of myelin-derived cholesterol, making recovery from SCI more difficult (4). The formation of foamy macrophages results from the excessive intracellular presence of lipids and dysregulated intracellular lipid homeostasis. They develop a proinflammatory phenotype which can contribute to additional neurologic decline (5).

RNA modifications that are essential for development and regeneration have recently attracted attention. The m6A modification is the most abundant RNA base methylation. Previous studies have shown a strong association between m6A modification and multiple pathological status, such as peripheral nerve injury, spinal cord injury and brain injury (6–8). m6A methylation levels are finely regulated by interactions between m6A methyltransferases (writers), demethylases (erasers), and binding proteins (readers) (8). However, changes in m6A modifications in the white blood cells of patients with SCI have not yet been reported. We identified gene and m6A expression changes in WBC, which may provide new strategies for treating SCI.

A systematic analysis of SCI gene signatures is necessary to identify novel SCI biomarkers. This not only improves the prognosis of SCI, but also provides novel drug targets for SCI treatment. Recently, gene co-expression network analysis has been used to identify candidate genes associated with SCI. Connectivity among different candidate genes can also be evaluated using weighted gene co-expression network analysis (WGCNA). Based on a microarray dataset (GSE151371) extracted from the Gene Expression Omnibus (GEO) database, we constructed a gene co-expression network and m6A correlation to screen candidate genes involved in SCI. Our study suggests that RNA modulation could be a new therapeutic target for SCI.

## Result

### WGCNA module construction and selection of modules with highly correlated with SCI

**Figure 1:**
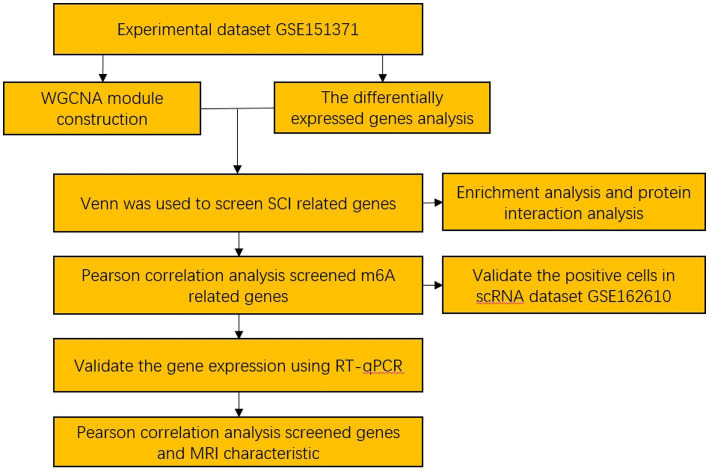
The workflow for prognostic analysis of m6A-related genes as potential biomarkers for spinal cord injury.

**Figure 2:**
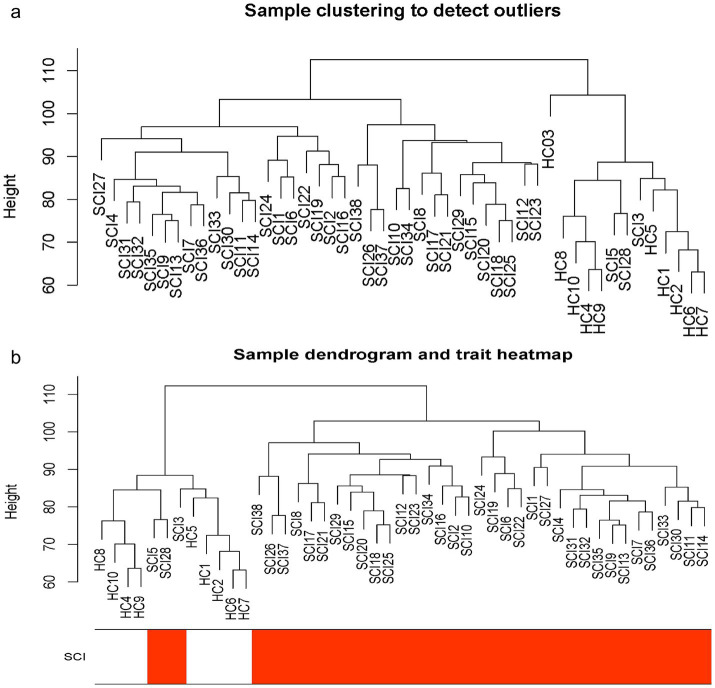
The cluster analysis. **(a)** Sample clustering diagram (delete 1 outlier samples by setting the height to 100). **(b)** Sample clustering diagram with clinical features. SCI, spinal cord injury; WGCNA, weighted gene co-expression network analysis.

**Figure 3:**
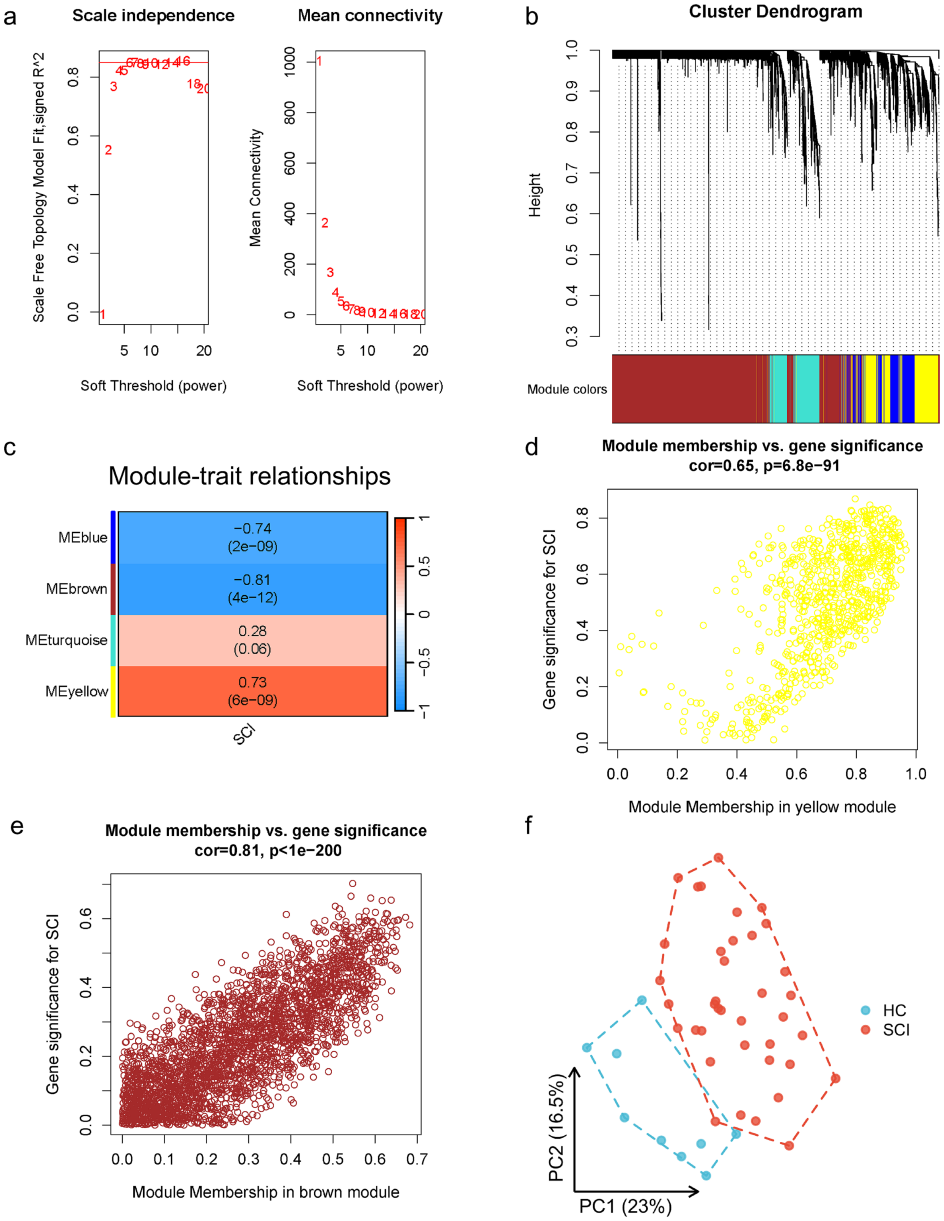
WGCNA module construction and selection of modules with high correlation with SCI. **(a)** Determination of the optimal soft threshold (in the process of module selection, the adjacency matrix is converted into a topology matrix, and the optimal soft threshold *β* = 16 is determined). **(b)** Cluster tree of co-expressed gene modules (similar genes are grouped into the same module through dynamic splicing and cluster analysis). **(c)** The correlation between gene modules and clinical information (the redder the colour, the higher the positive correlation; the greener the colour, the higher the negative correlation. Numbers in the figure are Pearson’s correlation coefficient, and corresponding *p*-values are in parentheses). **(d,e)** The correlation between black and pink modules and SCI is represented by scatter plot. **(f)** PCA (principal component analysis) of two group (blue are healthy control, red are SCI).

The workflow of this study is shown in Figure 1. WGCNA was performed using a gene expression matrix. After setting a high degree, one outlier samples (GSM4576310, Healthy Control 3) were removed (Figure 2a). Finally, 38 SCI and nine normal samples were analysed (Figure 2b). When the scale-free topological fitting index R2 reached 0.9, the appropriate chosen *β* value was 16 (Figure 3a). A dynamic clipping tree algorithm was used to segment the modules and construct a network diagram. Cluster analysis was performed on the modules, and modules with similarity greater than 0.5 were merged into new modules, in which the minimum module had 30 genes and the clipping height was 0.5 (Figure 3b). The WGCNA, based on a sequence-free network, was used to modularize genes, the topological overlap matrix between all genes was described by a heat map, and the relationship between sample features and modules was analysed. The colours corresponding to the modules are blue, brown, turquoise, and yellow. *BCL11B*, *UGT2B28*, *POLE2*, and *FCAR* were identified as key genes in each module by selecting the top hub in each module function. Among them, the gray module was the gene that cannot be clustered with other modules; therefore, it was not analysed in the subsequent analysis (Figure 3c). Key modules were identified according to the correlation coefficients between module features and traits, in which the yellow module had the highest positive correlation (cor = 0.65, *p* < 6.8 × 10^−81^), the brown module had the highest negative correlation (cor = 0.81, *p* < 1 × 10^−200^), and the yellow and brown modules had the highest degrees of SCI correlation. A scatter plot was used to represent the correlation between the yellow or brown modules and SCI, and 3,311 genes were identified (Figures 3d,e).

The differentially expressed genes (DEGs) between SCI and normal samples were screened.

**Figure 4:**
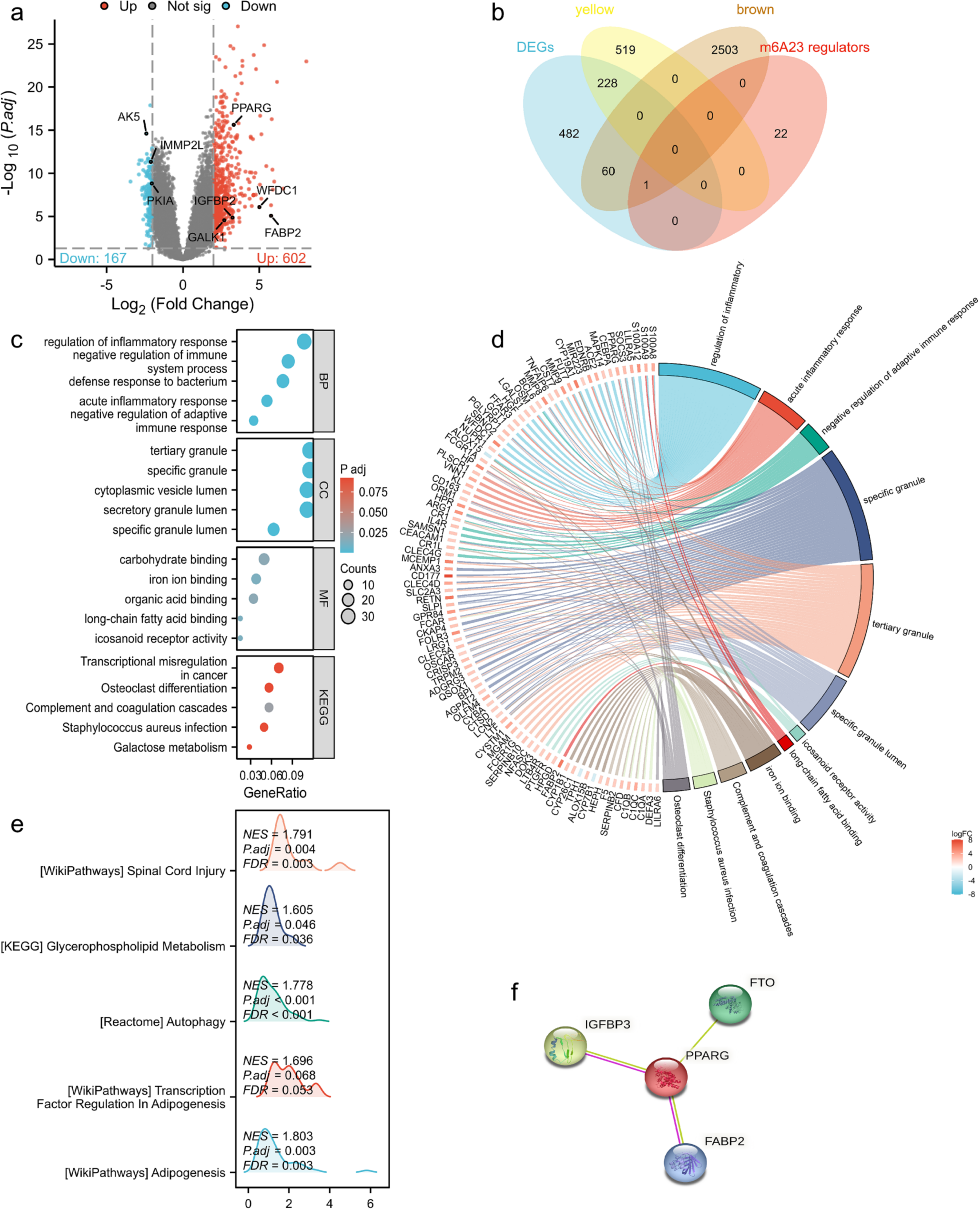
The DEGs and enrichment analysis of the SCI. **(a)** Volcano map of DEGs (red are up-regulated genes, blue are down-regulated genes, gray are non-DEGs). **(b)** A Venn diagram was utilized to screen the hub genes between the DEGs and WGCNA. Two hundred and eighty-nine genes were screened as candidates for further analysis and validation; GO and KEGG enrichment analysis were performed for SCI-highly correlated DEGs. **(c)** Bubble pattern. **(d)** Chord diagram. **(e)** GSEA. **(f)** Protein–protein interaction. The KEGG database was used with permission (28). DEG, differentially expressed gene; CI, spinal cord injury; WGCNA, weighted gene co-expression network analysis.

Principal component analysis (PCA) showed that the two groups were distinguishable (Figure 3f). Using the limma package in R language to screen DEGs based on|log2FC| >2 and *p* < 0.05, the differential genes in the SCI and healthy samples in the GSE151371 dataset were screened. A total of 769 DEGs were identified, of which 602 were upregulated and 167 were downregulated. The DEGs were used to construct a volcano plot, where red represents upregulated genes, blue represents downregulated genes, and gray represents non-differential genes (Figure 4a).

### Screening of SCI-highly correlated DEGs

There were 747 genes in the yellow and 2,564 genes in the brown modules obtained using WGCNA that were highly correlated with SCI, and the 769 genes obtained by DEG analysis were significantly different. There were 228 DEGs in the yellow module and 61 DEGs in the brown module. Therefore, 289 genes were obtained by taking the intersection of the two genes through a Venn diagram, and these genes were defined as SCI-highly correlated DEGs (Figure 4b). Among *BCL11B*, *UGT2B28*, *POLE2*, and *FCAR*, only FCAR was significantly differentially expressed. Among the m6A regulators, only *IGFBP2* was significantly differentially expressed in the brown module.

### The enrichment analysis of the SCI-highly correlated DEGs

The results showed that the candidate genes mainly focused on the biological processes of regulation of inflammatory response, negative regulation of immune system process, and molecular functions of carbohydrate, iron ion, organic acid, and long-chain fatty acid binding (Figures 4c,d). GSEA showed that these genes were mainly involved in glycerophospholipid metabolism, autophagy, SCI, adipogenesis, and transcription factor regulation in adipogenesis (Figure 4e). The relationship between lipid metabolism-related genes and m6A regulatory proteins was further analysed using protein–protein interactions (PPI) (Figure 4f).

### Screening and enrichment analysis of m6A-related candidate genes

**Figure 5:**
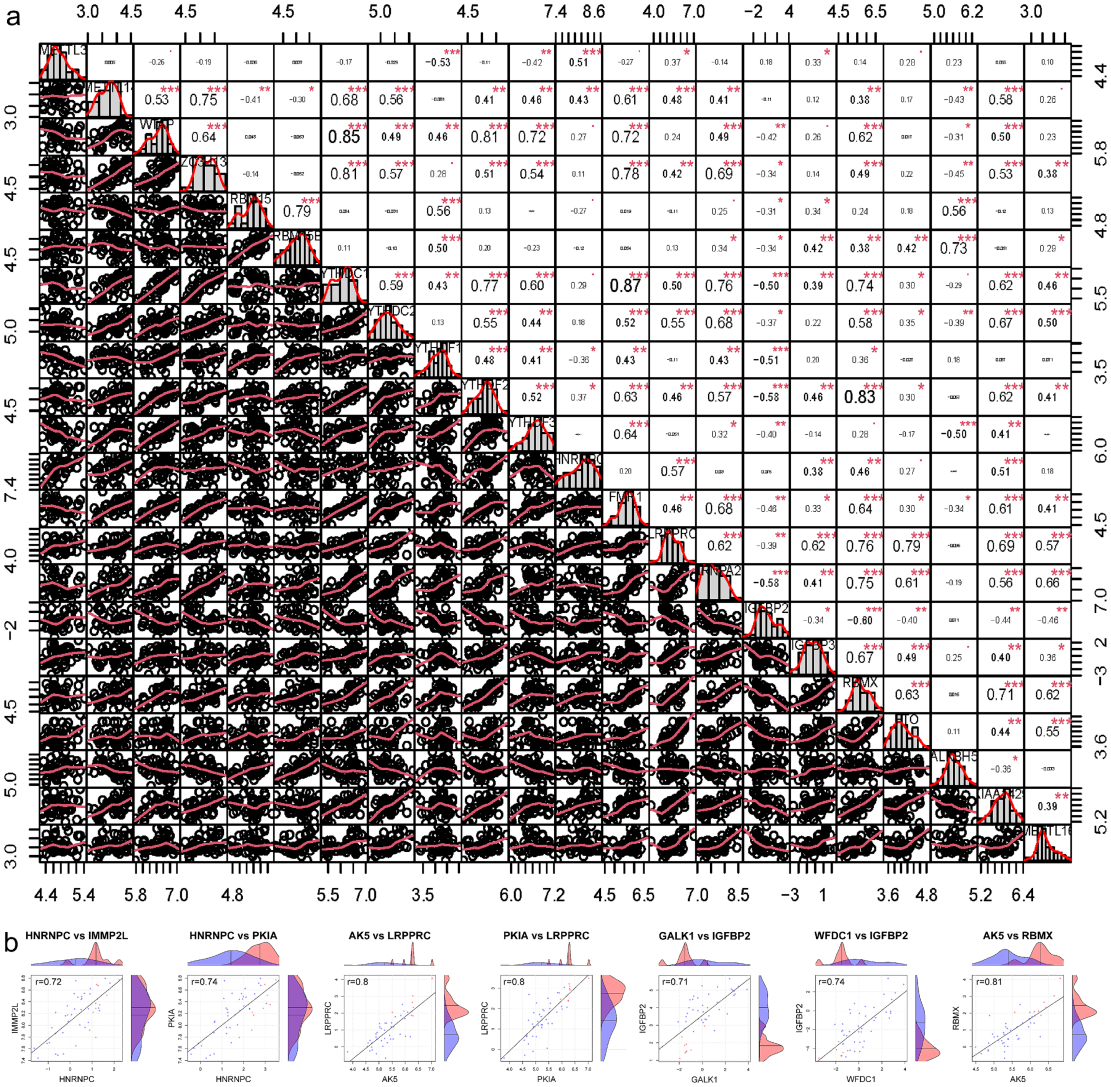
Screening m6A related candidate genes. **(a)** The correlation between the expression levels of 21 m6A regulators. **(b)** Pearson correlation analysis was used to screen out m6A-related candidate genes in SCI. SCI, spinal cord injury.

Lacking of IGFBP1 expression data, the correlation between the expression levels of 22 m6A regulators were calculated (Figure 5a). Pearson correlation analysis was used to screen five candidate genes (*IMMP2L*, *PKIA*, *AK5*, *GALK1*, and *WFDC1*) related to m6A in SCI that were highly correlated DEGs (|Pearson *R*| >0.7, *p* < 0.05) (Figure 5b).

### scRNA-seq analysis in mice SCI

After integrating data from the sham (10,227 cells), 1 day post injury (dpi) (12,783 cells), 3 dpi (16,447 cells), and 7 dpi groups in the GSE162610 mice dataset, a total of 39,457 cells were identified.

**Figure 6:**
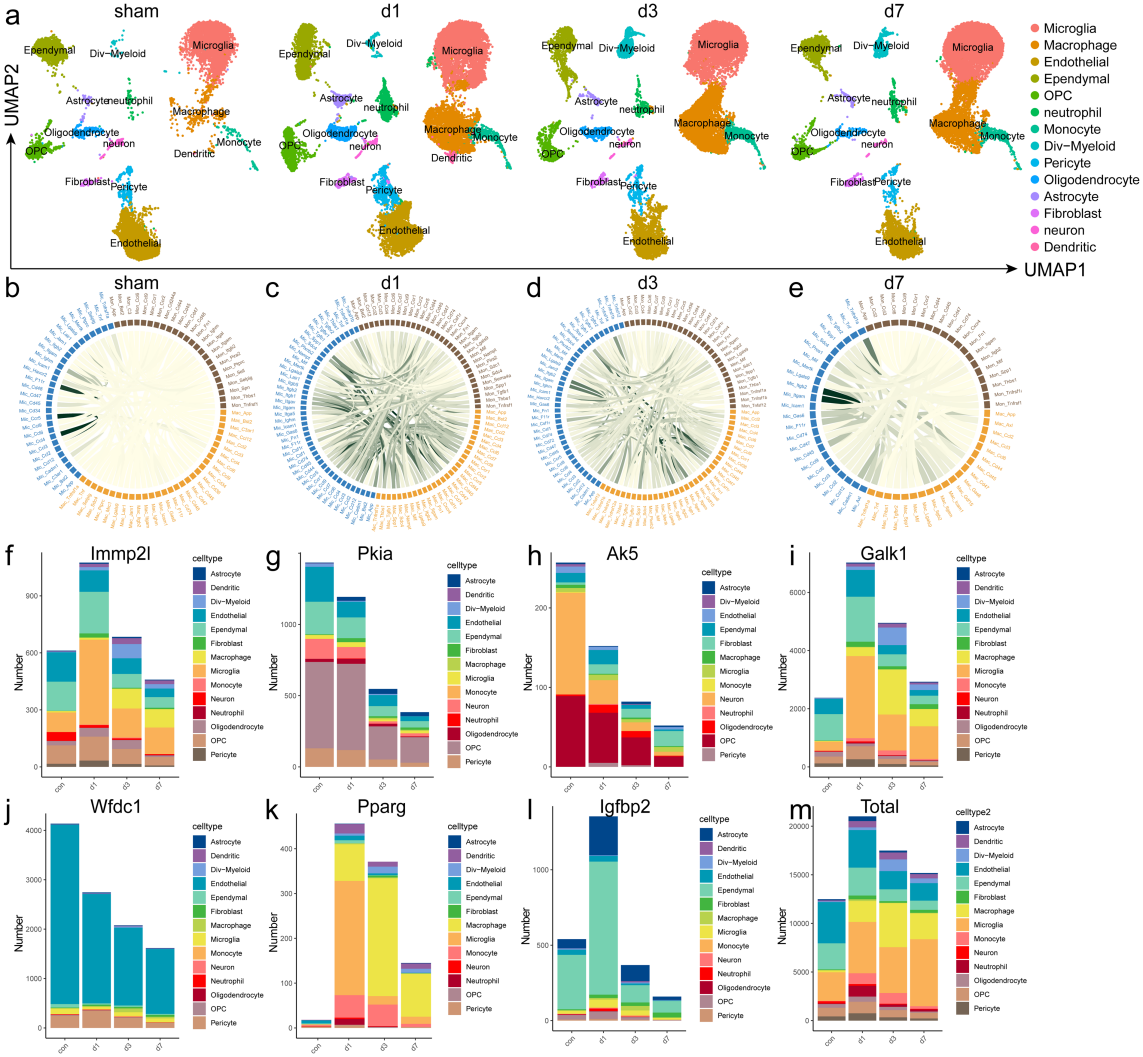
scRNA-seq analysis. **(a)** UMAP plots of SCI mice split by time point. **(b–e)** CellChat analysis reveals that WBC-dependent cell–cell communication (CCC). Chord plots showing interactions between monocyte (brown), macrophage (blue) and lymphocyte (orange) in sham, 1 dpi, 3 dpi, and 7 dpi spinal cords from vehicle and SCI animals. CCC was almost absent in the sham group, gradually weakened with the progression of days post injury. The strength of the interaction is indicated by the CCC score. The score is defined as the probability of CCCs by integrating gene expression data with prior database knowledge of the interactions between signalling ligands, receptors, and their cofactors. **(f–m)** scRNA-seq revealed different number of positive cells and the total number of cells in each group. SCI, spinal cord injury.

Peripheral immune cell infiltration began at 1dpi and reached a peak at 3 dpi (Figures 6a,m).

We verified the positive cell numbers and changes in eight candidate genes in a single-cell dataset. However, *FABP2* data were missing from this dataset; therefore, we validated the remaining seven genes (*IMMP2L*, *PKIA*, *AK5*, *GALK1*, *WFDC1*, *PPARG*, and *IGFBP2*) (Figures 6f–l). The trend in the number of positive cells was consistent with that of the peripheral gene expression. However, some changes were not completely consistent, which may have been due to differences in the periphery and injury site.

### CellChat of macrophage, microglia and monocytes in mice SCI

To quantitatively infer and analyse cell communication networks after SCI, we computationally isolated microglia, macrophages, and monocyte clusters from scRNAseq datasets and used CellChat to extract complex signalling patterns involving soluble and membrane-bound ligand-receptor interactions (9). “Chord plots” in Figures 6b–e show autocrine and paracrine signalling interactions with colour-coded ligandreceptor interaction scores in each condition. Cell–cell communication (CCC) was almost absent in the sham group, significantly increased at 1 dpi and gradually weakened with the progression of days post injury. This suggests that WBC infiltration in the early stage of SCI has a substantial effect on the spinal cord microenvironment.

### Validating the correlation between expression level and prognosis of m6A-related candidate genes

Clinical blood samples were used to validate the gene (*IMMP2L*, *PKIA*, *AK5*, *GALK1*, *WFDC1*, *PPARG*, *FABP2*, and *IGFBP2*) expression by RT-qPCR (Figure 6a). We assessed the maximum canal compromise (MCC) and maximum spinal cord compression (MSCC) using magnetic resonance imaging (MRI; Figure 6b). In the correlation analysis of the genes and MRI characteristics, the WBC count of AK5 (cor = −0.656, *p* = 0.0396) was significantly correlated with MCC, and PPARG (cor = 0.84, *p* = 0.0022) was significantly correlated with MSCC (Figures 6c–j). These results indicated that AK5 and PPARG were associated with the degree of spinal cord compression.

## Discussion

It is particularly important to identify effective treatments and prognostic targets in the early stages of injury. In this study, early spinal cord injury was accompanied by significant immune cell infiltration. The single-cell results suggest that the lesion site was accompanied by significant immune infiltration during the early stage of spinal injury.

A total of 289 genes were obtained from the intersection of the DEG and module genes with the highest correlation in the selected WGCNA.

m6A is the most abundant post-transcriptional mRNA in most eukaryotes (10). It is involved in various cellular processes, including neurosciences (11). RMVar analysis showed that all five key genes (*IMMP2L*, *PKIA*, *AK5*, *GALK1*, and *WFDC1*) contained m6A sites, which supports our results (12).

The 289 DEGs showed were mainly enriched in pathways, such as long-chain fatty acid binding and transcription factor regulation in adipogenesis. PPARG and FABP2 play key roles in lipid metabolism.

Myelin contains large amounts of lipids (approximately 80% of the dry weight) (13). It is crucial for macrophages to efficiently clear excess myelin debris to prevent the peroxidation of myelin-derived lipids at the lesion site after SCI (5). However, dysregulated mechanisms of lipid autophagy or efflux often result in the persistence of lipids in macrophages after the phagocytosis of myelin debris (14). This dysregulation leads to the formation of foam cells, characterised by excessive intracellular lipid accumulation and disrupted intracellular lipid homeostasis, which is typically observed a week after SCI. Therefore, whether further PPARG overexpression can promote lipid metabolism and reduce lipid accumulation after injury needs to be addressed.

Macrophages play a vital role in the clearance of myelin fragments (15), with CD68^+^/CD163^−^ macrophages continuing to phagocytose myelin at the lesion site beyond 16 weeks in post-SCI models (16, 17).

The prolonged phagocytic process may eventually result in foam cell formation, triggering cytokine release and ultimately leading to neuronal apoptosis. By directly or indirectly stimulating the lipid efflux transporters, ABCA1 and ABCG1, PPARG prevents foam cell formation and promotes remyelination by reversing cholesterol transport to the injured spinal cord. Therefore, PPARG is a promising therapeutic target for SCI treatment.

After SCI, PPARG, ABCA1, and ABCG1 expression significantly increased at the lesion site, which improved lipid efflux and led to a reduction in foam cell formation. High expression of these genes has an important protective effect on cells. Notably, this effect was extrapolated to an acute SCI model *in vivo* and found to be associated with improved functional outcomes, emphasising that ABCA1 and ABCG1 are promising targets for SCI treatment. Therefore, it is important to target the foam cell formation processes to promote lipid efflux and prevent harmful pro-inflammatory cytokine storms. One promising therapeutic target is PPARG, which can prevent foam cell formation and promote remyelination by stimulating the lipid efflux transporters ABCA1 and ABCG1 (18). Similarly, atorvastatin may reduce injury and improve recovery after SCI by activating PPARG and, consequently, ABCA1 (19). Notably, increased expression of PPARG, ABCA1, and ABCG1 at the lesion site after SCI significantly improved lipid efflux, offering crucial cellular protection and demonstrating its potential as a therapeutic approach. Thus, the indirect upregulation of ABCA1 via PPARG activation could make PPARG a promising therapeutic target for stimulating the lipid efflux of foamy macrophages in SCI (20, 21).

Targeting the phagocytic process can positively affect SCI recovery through various strategies. However, stimulating phagocytic receptors, which may turn macrophages into foam cells, negatively impacts functional recovery after SCI (22).

Furthermore, the genetic deletion of CD36, a phagocytic receptor, led to decreased lipid uptake and improved functional recovery in mice with SCI, suggesting that myelin clearance alone may not always be optimal. Therefore, targeting the stimulation of the lipid efflux transporters ABCA1 and ABCG1, directly or indirectly via PPARG, can be an effective approach to combat foam cell formation in SCI. Additionally, stimulation of reversed cholesterol transport has been proven sufficient to restore the capacity to remyelinate lesioned tissues (4).

High PPARG expression correlates with worse prognosis in patients with SCI, whereas low expression of AK5 predicts worse prognosis. AK5 is significantly correlated with m6A regulators (LRPPRC and RBMX). This regulation can be direct or indirect; however, the specific mechanisms remain unknown.

Notably, the number of PPARG-and AK5-positive cells showed the same trend as peripheral gene expression. The numbers of PPARG-positive macrophages and microglia significantly increased in the early stages of SCI, confirming that PPARG plays a key role in the early stages of injury.

Future research will focus on regulating the role of PPARG and AK5 on lipid metabolism, reducing inflammation or promoting nerve repair in spinal cord injury. In the case of spinal cord injury, it may be feasible to develop treatments that target m6A modifications or these specific genes. Further studies will focus on m6A regulatory enzymes to investigate whether key m6A related enzymes are changed in SCI and how they participate in the regulation of PPARG and AK5 expression.

In conclusion, our findings suggested that these two genes are closely associated with SCI progression. However, studies on the role of PPARG and AK5 in SCI are rare. These results indicated that the key genes screened by bioinformatics methods were highly correlated with the occurrence and development of SCI, and significantly correlated with the prognosis of SCI patients. Therefore, these two key genes may serve as references for the prognosis and treatment of SCI.

However, this study had certain limitations. First, our results are based on data from existing public databases and small clinical samples. Therefore, a large-scale, prospective, multicentre study is required to validate our results. Second, the study population was primarily Chinese. Therefore, our findings may not be optimal for patients from other countries or ethnicities. Third, the correlation between key genes and the development and progression of SCI has not yet been confirmed through biological experiments. In follow-up studies, experimental validation will be performed to reveal the relationship between the key genes and SCI. Thus, we determined their suitability as new prognostic and therapeutic targets to provide a rationale for the clinical prognosis and treatment of SCI.

## Methods

### Data collection and processing

We searched the GEO database^1^ for keywords, such as “spinal cord injury,” “white blood cell,” and “blood.” We included the GSE1513171 dataset as the experimental dataset in the study, which contains gene expression profiles of peripheral WBCs and the corresponding clinical data of 38 patients with SCI and 10 healthy individuals. The probes were converted to the corresponding gene symbols by referring to the annotation information of the GPL20301 [Illumina HiSeq 4000 (*Homo sapiens*)] platform.

### Construction of WGCNA

To explore the modules and genes related to the clinical characteristics of healthy individuals and patients with SCI, the data from GSE151371 were analysed using the WGCNA package of the R language, and the samples were clustered. To ensure the reliability of the results, we analysed the samples and removed samples that were not clustered, that is, outlier samples (23). To ensure that the network conforms to the scale-free network distribution, the “pick Soft Threshold” function in the WGCNA package was used to calculate the correlation coefficient of *β* value and the mean of gene connectivity, and the appropriate soft threshold β is selected to make the network conform to the standard of scale-free network (24). The modules were then clustered with a minimum of 30 genes and a cut height of 0.5. Gene significance and module membership were calculated and correlated with clinical traits. Two modules with the highest correlation with SCI were selected and the genes in these modules were further analysed. Genes in the co-expression module have high connectivity and genes in the same module may have similar biological functions.

### DEG analysis

PCA was used to determine the significant differences with *p* < 0.05 (25). The R language (R) 4.0.3 limma package was used to analyse gene differences between the gene expression matrix of peripheral blood monocytes of healthy individuals and patients with SCI. The screening criteria were set as |log2FC| >2 and *p* < 0.05, the correction method was FDR, and (26) upregulated and downregulated genes were identified using volcanoes maps.

### Screening of DEGs highly associated with SCI

The common genes obtained by WGCNA and DEG analyses were defined as SCI that highly correlated with differential genes. A Venn diagram was used to show all the DEGs associated with SCI.

### Gene function and pathway enrichment analysis

The online website DAVID^2^ was used to analyse the module function and pathway enrichment of m6A-related candidate genes to further explore the biological functions of these genes (27). GO analysis was used to annotate the functions of the genes and their products in three aspects: biological processes, molecular functions, and cellular components. KEGG database is a collection of information about genes, proteins, chemical components and their interactions, reactions and relationship networks to annotate gene functions and metabolic pathways (28). We uploaded all identified genes to the Search Tool for the Retrieval of Interacting Genes/Proteins database to construct a protein–protein interaction (PPI) network (29).

### Identification of m6A-related candidate genes

Cor () and cor. test () functions of R language were used to calculate the correlation between the expression levels of 23 m6A regulators (METTL3, METTL14, METTL16, WTAP, VIRMA, ZC3H13, RBM15, RBM15B, YTHDC1, YTHDC2, YTHDF1, YTHDF2, YTHDF3, HNRNPC, FMR1, LRPPRC, HNRNPA2B1, IGFBP1, IGFBP2, IGFBP3, RBMX, FTO, and ALKBH5) and the expression levels of SCI-highly correlated differential genes and calculate the *p*-value (8). Genes significantly associated with m6A regulators (|Pearson *R*| > 0.7 and *p* < 0.05) were identified as candidate genes related to m6A (30).

### scRNA-seq data processing and identification using CellChat

For single-cell genomics analysis, the processed data were analysed using the “Seurat” package. The dimension-reduction method for single-cell visualisation is uniform manifold approximation and projection. Finally, the CCC was calculated using “CellChat” (31).

### Patients and clinical data

We recruited 10 patients with SCI caused by acute trauma at the First Affiliated Hospital of Nanjing Medical University between 2022 and 2023. All patients were neurologically assessed by certified orthopaedic surgeons. Neurological assessments including sensory, motor, and reflex examinations below the injured segment were performed according to the American Spinal Injury Association (ASIA) Impairment scale (32). The inclusion criteria were as follows: (1) trauma-induced acute SCI between C2 and L1; (2) obvious symptoms of paraplegia or quadriplegia, assessed as ASIA grades A–C; (3) eye opening of 4 and verbal response of 6 according to the Glasgow Coma Scale; (4) age >18 years; (5) abnormal spinal cord signal detected using MRI; and (6) completion of effective and reliable neurological function tests. The exclusion criteria were as follows: (1) ASIA scores of D and E, (2) MRI showing no definite changes in spinal cord signalling, (3) combination of severe craniocerebral injury and intracranial hypertension, and (4) history of tumour or autoimmune disease.

Ten patients undergoing intrathecal anaesthesia (with non-neurological or neoplastic diseases) were recruited as controls. The control group was aged >18 years and showed no obvious abnormalities after neurological evaluation. Patients with nervous system diseases, tumours, rheumatic diseases, or fractures were excluded. All patients provided informed consent, and third-party assent was not allowed. The study protocol was approved by the Ethics Committee of the First Affiliated Hospital of Nanjing Medical University (2022-SR-697). We identified a committee that approved the research and confirmed that it was performed in accordance with the relevant guidelines and regulations, confirming that informed consent was obtained from all participants and/or their legal guardians. Whole blood was collected at–24–48 h after SCI. Two millilitres of blood was drawn each time, and the WBCs were collected by adding red blood cell lysate after centrifugation at 3,000 × g for 5 min.

### RNA isolation and real-time PCR

For expression analysis, WBC from patients was isolated using Red Blood Cell Lysis Buffer (BL503A, Biosharp). Total RNA was isolated according to the manufacturer’s instructions (R6834-02, Omega), and RNA concentration and quality were measured using a NanoDrop spectrophotometre (Thermo Fisher Scientific). After cDNA synthesis using PrimeScript RT Master Mix (R222, Vazyme), real-time PCR was performed using SYBR qPCR Master Mix (Q341, Vazyme) on a QuantStudio 7 Flex Real-Time PCR System (Thermo Fisher Scientific). All primers used are listed in Table 1. Using the ΔΔC_T_ method, the expression levels of target genes were calculated (*GAPDH* as a housekeeping gene, healthy control group as reference samples).

**Table 1:** Primer sequences.

IMMP2L	F: cct ctc cca gtc ctc aca
IMMP2L	R: acc tgc cca aca ctt
PKIA	F: ttt aca ata acg agc cca ctt
PKIA	R: gca caa cca cac aaa tta cc
AK5	F: gtt gcc cag gct cta tct t
AK5	R: tgt tct gca cgc ttc agt
GALK1	F: acc tca tcg ggg aac aca c
GALK1	R: gag aga cac cag ccc atc c
WFDC1	F: aga tgg gcg aat cct acg
WFDC1	R: tgc ttg ccg ttg ctt tac
PPARG	F: gcc tgc atc tcc acc tt
PPARG	R: tcc aca gac acg aca ttc a
FABP2	F: tgc caa agc aaa aga agt aaa
FABP2	R:tat aaa gca aca tgg acg ca
IGFBP2	F: agc ccc tca agt cgg gta t
IGFBP2	R: ggg tgg tcg cag ctt ctt
GAPDH	F: ggg gct ctc cag aac atc
GAPDH	R: tga cac gtt ggc agt gg

### MRI

All patients with SCI underwent MRI to assess the severity of their injuries. MCC and MSCC were used to measure spinal cord compression (33) (Figure 6b). Mid sagittal T2-weighted MRI images were used to identify MCC and MSCC. MCC and MSCC were calculated as follows:

$$MCC=\left[\left(\mathrm{Da}+Db\right)/2-Di\right]/\left(\mathrm{Da}+Db\right)/2\times 100$$

$$\mathrm{MSCC}=\left[\left(da+db\right)/2-di\right]/\left(da+db\right)/2\times 100$$

**Figure 7:**
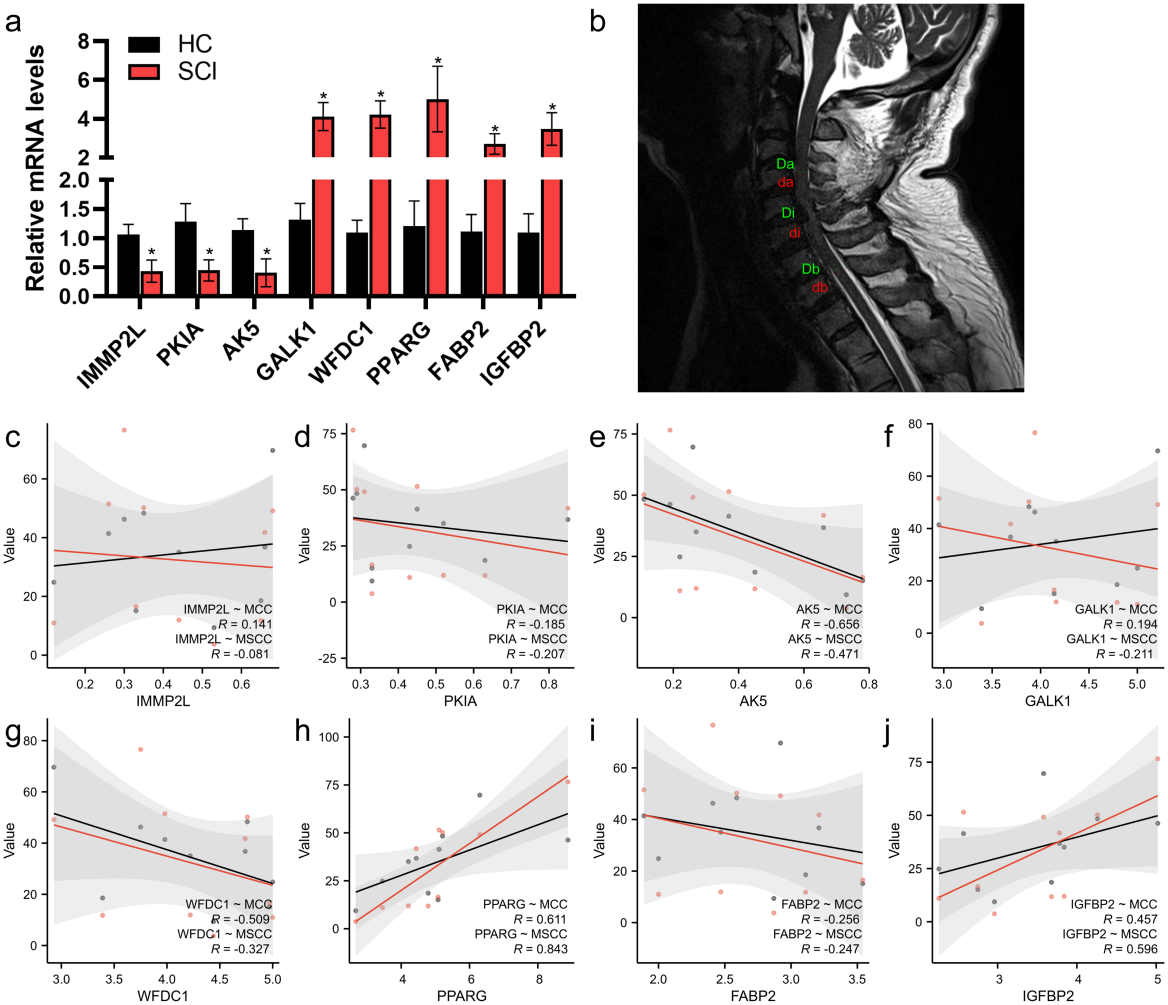
Screening of key genes associated with SCI diagnosis. **(a)** Gene expression levels between spinal cord injury (SCI) and healthy controls (HC). **(b)** Representative MRI of patients with SCI and measurement of MCC and MSCC (*n* = 10). **(c–j)** The relationship between genes and MCC/MSCC length. (Red are MCC correlation coefficient, black are MSCC correlation coefficient). ^*^p < 0.05. Pearson’s correlation test in **c–j**. MCC, maximum canal compromise; MSCC, maximum spinal cord compression; Di, midsagittal diameter of the spinal canal at the site of injury; Da, Db, the diameter of the spinal canal above/below the level of spinal injury; di, the diameter of the spinal cord at the level of SCI; da, db, the diameter of the spinal cord at a normal segment above/below the level of SCI.

Di represents the midsagittal diameter of the spinal canal at the site of injury; Da and Db represent the diameter of the spinal canal above/below the level of spinal injury; di represents the diameter of the spinal cord at the level of SCI; and da and db represent the diameter of the spinal cord at a normal segment above or below the level of SCI (34). To normalise the relative length of the SCIs, the vertebral heights at the upper and lower stages of the injured area were measured. Furthermore, genes significantly associated with MCC and MSCC (|Pearson *R*| >0.5 and *p* < 0.05) were identified as candidate genes associated with prognosis (see Figure 7).

### Statistical analysis

In this study, R (version 4.2.3) and R Studio software were used to perform statistical analysis and figure preparation. *p*-values less than 0.05 were defined as statistically significant.

## Data Availability

The datasets presented in this study can be found in online repositories. The names of the repository/repositories and accession number(s) can be found in the article/supplementary material.

## Footnotes

^1^ https://www.ncbi.nlm.nih.gov/geo/

^2^ https://david.ncifcrf.gov/

## References

[1] Eli I Lerner DP Ghogawala Z. Acute traumatic spinal cord injury. Neurol Clin. (2021) 39:471–88. doi: 10.1016/j.ncl.2021.02.004, PMID: 33896529

[2] Eckert MJ Martin MJ. Trauma: spinal cord injury. Surg Clin North Am. (2017) 97:1031–45. doi: 10.1016/j.suc.2017.06.008, PMID: 28958356

[3] Van Broeckhoven J Sommer D Dooley D Hendrix S Franssen A. Macrophage phagocytosis after spinal cord injury: when friends become foes. Brain. (2021) 144:2933–45. doi: 10.1093/brain/awab250, PMID: 34244729

[4] Cantuti-Castelvetri L Fitzner D Bosch-Queralt M Weil MT Su M Sen P. Defective cholesterol clearance limits remyelination in the aged central nervous system. Science. (2018) 359:684–8. doi: 10.1126/science.aan4183, PMID: 29301957

[5] Wang X Cao K Sun X Chen Y Duan Z Sun L. Macrophages in spinal cord injury: phenotypic and functional change from exposure to myelin debris. Glia. (2015) 63:635–51. doi: 10.1002/glia.22774, PMID: 25452166 PMC4331228

[6] Xing L Cai Y Yang T Yu W Gao M Chai R. Epitranscriptomic m6A regulation following spinal cord injury. J Neurosci Res. (2021) 99:843–57. doi: 10.1002/jnr.24763, PMID: 33271625

[7] Li C Zhao J Qin T Jin Y Duan C Wu T. Comprehensive analysis of m6A methylation modification in chronic spinal cord injury in mice. J Orthop Res. (2023) 41:1320–34. doi: 10.1002/jor.25457, PMID: 36205185

[8] Liu D Fan B Li J Sun T Ma J Zhou X. N6-methyladenosine modification: a potential regulatory mechanism in spinal cord injury. Front Cell Neurosci. (2022) 16:989637. doi: 10.3389/fncel.2022.989637, PMID: 36212687 PMC9539101

[9] Jin S Guerrero-Juarez CF Zhang L Chang I Ramos R Kuan CH. Inference and analysis of cell–cell communication using CellChat. Nat Commun. (2021) 12:1088. doi: 10.1038/s41467-021-21246-9, PMID: 33597522 PMC7889871

[10] Garbo S Zwergel C Battistelli C. m6A RNA methylation and beyond—the epigenetic machinery and potential treatment options. Drug Discov Today. (2021) 26:2559–74. doi: 10.1016/j.drudis.2021.06.004, PMID: 34126238

[11] Deng LJ Deng WQ Fan SR Chen MF Qi M Lyu WY. m6A modification: recent advances, anticancer targeted drug discovery and beyond. Mol Cancer. (2022) 21:52. doi: 10.1186/s12943-022-01510-2, PMID: 35164788 PMC8842557

[12] Luo X Li H Liang J Zhao Q Xie Y Ren J. RMVar: an updated database of functional variants involved in RNA modifications. Nucleic Acids Res. (2021) 49:D1405–d1412. doi: 10.1093/nar/gkaa811, PMID: 33021671 PMC7779057

[13] Dimas P Montani L Pereira JA Moreno D Trötzmüller M Gerber J. CNS myelination and remyelination depend on fatty acid synthesis by oligodendrocytes. eLife. (2019):e44702:8. doi: 10.7554/eLife.44702, PMID: 31063129 PMC6504237

[14] Zhu Y Lyapichev K Lee DH Motti D Ferraro NM Zhang Y. Macrophage transcriptional profile identifies lipid catabolic pathways that can be therapeutically targeted after spinal cord injury. J Neurosci. (2017) 37:2362–76. doi: 10.1523/JNEUROSCI.2751-16.2017, PMID: 28130359 PMC5354348

[15] Miron VE Boyd A Zhao JW Yuen TJ Ruckh JM Shadrach JL. M2 microglia and macrophages drive oligodendrocyte differentiation during CNS remyelination. Nat Neurosci. (2013) 16:1211–8. doi: 10.1038/nn.3469, PMID: 23872599 PMC3977045

[16] Kwiecien JM Dabrowski W Dąbrowska-Bouta B Sulkowski G Oakden W Kwiecien-Delaney CJ. Prolonged inflammation leads to ongoing damage after spinal cord injury. PLoS One. (2020) 15:e0226584. doi: 10.1371/journal.pone.0226584, PMID: 32191733 PMC7081990

[17] David S Greenhalgh AD Kroner A. Macrophage and microglial plasticity in the injured spinal cord. Neuroscience. (2015) 307:311–8. doi: 10.1016/j.neuroscience.2015.08.064, PMID: 26342747

[18] Lin XL Hu HJ Liu YB Hu XM Fan XJ Zou WW. Allicin induces the upregulation of ABCA1 expression via PPARγ/LXRα signaling in THP-1 macrophage-derived foam cells. Int J Mol Med. (2017) 39:1452–60. doi: 10.3892/ijmm.2017.2949, PMID: 28440421 PMC5428973

[19] Gao S Zhang ZM Shen ZL Gao K Chang L Guo Y. Atorvastatin activates autophagy and promotes neurological function recovery after spinal cord injury. Neural Regen Res. (2016) 11:977–82. doi: 10.4103/1673-5374.184498, PMID: 27482228 PMC4962597

[20] Bensinger SJ Tontonoz P. Integration of metabolism and inflammation by lipid-activated nuclear receptors. Nature. (2008) 454:470–7. doi: 10.1038/nature07202, PMID: 18650918

[21] Sengupta MB Saha S Mohanty PK Mukhopadhyay KK Mukhopadhyay D. Increased expression of ApoA1 after neuronal injury may be beneficial for healing. Mol Cell Biochem. (2017) 424:45–55. doi: 10.1007/s11010-016-2841-8, PMID: 27734225

[22] Milich LM Ryan CB Lee JK. The origin, fate, and contribution of macrophages to spinal cord injury pathology. Acta Neuropathol. (2019) 137:785–97. doi: 10.1007/s00401-019-01992-3, PMID: 30929040 PMC6510275

[23] Langfelder P Zhang B Horvath S. Defining clusters from a hierarchical cluster tree: the dynamic tree cut package for R. Bioinformatics. (2008) 24:719–20. doi: 10.1093/bioinformatics/btm563, PMID: 18024473

[24] Horvath S Dong J. Geometric interpretation of gene coexpression network analysis. PLoS Comput Biol. (2008) 4:e1000117. doi: 10.1371/journal.pcbi.1000117, PMID: 18704157 PMC2446438

[25] Ma S Dai Y. Principal component analysis based methods in bioinformatics studies. Brief Bioinform. (2011) 12:714–22. doi: 10.1093/bib/bbq090, PMID: 21242203 PMC3220871

[26] Ritchie ME Phipson B Wu D Hu Y Law CW Shi W. Limma powers differential expression analyses for RNA-sequencing and microarray studies. Nucleic Acids Res. (2015) 43:e47. doi: 10.1093/nar/gkv007, PMID: 25605792 PMC4402510

[27] Sherman BT Hao M Qiu J Jiao X Baseler MW Lane HC. DAVID: a web server for functional enrichment analysis and functional annotation of gene lists (2021 update). Nucleic Acids Res. (2022) 50:W216–21. doi: 10.1093/nar/gkac194, PMID: 35325185 PMC9252805

[28] Kanehisa M Furumichi M Sato Y Matsuura Y Ishiguro-Watanabe M. KEGG: biological systems database as a model of the real world. Nucleic Acids Res. (2025) 53:D672–7. doi: 10.1093/nar/gkae909, PMID: 39417505 PMC11701520

[29] Szklarczyk D Gable AL Nastou KC Lyon D Kirsch R Pyysalo S. The STRING database in 2021: customizable protein-protein networks, and functional characterization of user-uploaded gene/measurement sets. Nucleic Acids Res. (2021) 49:D605–12. doi: 10.1093/nar/gkaa1074, PMID: 33237311 PMC7779004

[30] Wang Z Shen L Wang J Huang J Tao H Zhou X. Prognostic analysis of m6A-related genes as potential biomarkers in idiopathic pulmonary fibrosis. Front Genet. (2022) 13:1059325. doi: 10.3389/fgene.2022.1059325, PMID: 36523766 PMC9744785

[31] Brennan FH Li Y Wang C Ma A Guo Q Li Y. Microglia coordinate cellular interactions during spinal cord repair in mice. Nat Commun. (2022) 13:4096. doi: 10.1038/s41467-022-31797-0, PMID: 35835751 PMC9283484

[32] El Masry WS Tsubo M Katoh S El Miligui YH Khan A. Validation of the American Spinal Injury Association (ASIA) motor score and the National Acute Spinal Cord Injury Study (NASCIS) motor score. Spine. (1996) 21:614–9. doi: 10.1097/00007632-199603010-00015, PMID: 8852318

[33] Fehlings MG Furlan JC Massicotte EM Arnold P Aarabi B Harrop J. Interobserver and intraobserver reliability of maximum canal compromise and spinal cord compression for evaluation of acute traumatic cervical spinal cord injury. Spine. (2006) 31:1719–25. doi: 10.1097/01.brs.0000224164.43912.e6, PMID: 16816769

[34] Huang Y Gao P Qin T Chu B Xu T Yi J. Delayed inhibition of collagen deposition by targeting bone morphogenetic protein 1 promotes recovery after spinal cord injury. Matrix Biol. (2023) 118:69–91. doi: 10.1016/j.matbio.2023.03.006, PMID: 36918086

